# Editorial: *Acidobacteria* – Towards Unraveling the Secrets of a Widespread, Though Enigmatic, Phylum

**DOI:** 10.3389/fmicb.2022.960602

**Published:** 2022-06-24

**Authors:** Katharina J. Huber, Michael Pester, Stephanie A. Eichorst, Acacio A. Navarrete, Bärbel U. Foesel

**Affiliations:** ^1^Department Microorganisms, Leibniz Institute DSMZ - German Collection of Microorganisms and Cell Cultures, Braunschweig, Germany; ^2^Institute for Microbiology, Technical University of Braunschweig, Braunschweig, Germany; ^3^Department of Microbiology and Ecosystem Science, Centre for Microbiology and Environmental Systems Science, University of Vienna, Vienna, Austria; ^4^Graduate Program in Environmental Sciences, University Brazil, São Paulo, Brazil; ^5^Working Group Biobank, Research Unit Molecular Epidemiology, Institute of Epidemiology, Helmholtz Munich, German Research Center for Environmental Health, Neuherberg, Germany

**Keywords:** *Acidobacteriota*, phototrophy, phosphorus removal, niche adaptation, drought resistance, host-association, thermophile

The bacterial phylum, *Acidobacteriota* (Oren and Garrity, 2021) (formerly *Acidobacteria*) is a diverse and ubiquitous group of environmental bacteria originally discovered in the late 1990s (Kuske et al., 1997; Ludwig et al., 1997). Its members are prevalent in terrestrial environments, being the third most abundant soil phylum (Delgado-Baquerizo et al., 2018). Typically found in aquatic and terrestrial systems, they also occur in harsh environments such as acidic mine drainage (Kishimoto et al., 1991; Kleinsteuber et al., 2008; Falagán et al., 2017), rock faces (Zimmermann et al., 2006; Kelly et al., 2010; Marnocha and Dixon, 2014) or desert soil (Kuske et al., 1997; Dunbar et al., 2002). Despite their ubiquity and high 16S rRNA gene sequence abundance and diversity especially in soil habitats (Barns et al., 1999; Janssen, 2006; Fierer, 2017), knowledge on the ecology of *Acidobacteriota* is still surprisingly fragmentary, not least due to challenges in cultivating representative strains.

Nevertheless, the development of adapted cultivation media and techniques (Sait et al., 2002; Joseph et al., 2003; Davis et al., 2005; George et al., 2011), specific screening methods (Stevenson et al., 2004; Pankratov et al., 2008; Oberpaul et al., 2020), in addition to the rise of cultivation-independent, molecular approaches have provided insights into some key characteristics of single isolates and acidobacterial communities. Based on existing isolates with validated names and 16S rRNA database sequences from cultivation-independent studies, a provisional taxonomic system has been established, currently comprising 15 class-level units, only five of which contain described members (Dedysh and Yilmaz, 2018), namely the already earlier proposed classes “Acidobacteriae” (Oren et al., 2015), *Blastocatellia* (Pascual et al., 2015) and *Holophagae* (Fukunaga et al., 2008), as well as the two newly proposed *Vicinamibacteria* and *Thermoanaerobaculia*. As such, further efforts are needed to better understand the acidobacterial diversity, phylogeny and taxonomy, functional roles and reasons for the success of *Acidobacteriota* in the environment.

In this Research Topic further insights in diversity, ecology and evolution of the *Acidobacteriota* are presented in six original research articles. They touch topics as general as nutrient-cycling (Kristensen et al.) or ecological theories (Sikorski et al.), but also as specific as wastewater-treatment (Kristensen et al.), drought-resistance (Huber et al.) or host-association (Wang et al.). They not only span a variety of habitats from aquatic to terrestrial environments, but also provide insights into previously (Huber et al.; Saini et al.; Wang et al.) and newly (Kristensen et al.; Ruhl et al.; Wang et al.) discovered members of the phylum - from single species to whole clades.

Various edaphic properties have been investigated to better understand the factors that govern acidobacterial abundance and diversity in soils. More specifically, soil pH (Jones et al., 2009; Lauber et al., 2009), factors linked to soil acidity (Navarrete et al., 2015), carbon content (Eichorst et al., 2011), carbon and nitrogen content (Ivanova et al., 2020) and nutrient availability (Navarrete et al., 2015; de Chaves et al., 2019) have been identified as major determinants, showing both, positive and negative correlations to different taxa. Here Huber et al. expand on those observations and explore water limitation as one of the major factors potentially impacting *Acidobacteriota* in/from subtropical savannah soils by RNA-based community screening and physiological testing. Sikorski et al. use a culture-independent niche modeling approach to elucidate ecological adaptations and evolution of acidobacteria in German grassland soils. As seen earlier e.g., by Naether et al. (2012); Foesel et al. (2014), both showed group or even species-specific response or adaptation, respectively.

(Meta)genomic investigations of the *Acidobacteriota* have provided tremendous insights into their potential role(s) in the environment. Since the first genome data became available (Ward et al., 2009), *Acidobacteriota* have been postulated to play a role in global C- and N-cycling. Subsequent investigations have collaborated these postulates with further physiological and genomic evidence in regards to organic carbon degradation (Rawat et al., 2012; Kielak et al., 2016; Eichorst et al., 2018) and participation in the N-cycle via reduction of nitrate to nitrite in select isolates (Männistö et al., 2012; Huber et al., 2017). Additional “-omic” investigations, many of which were combined with physiological investigations, have identified select *Acidobacteriota* to be phototrophic (Bryant et al., 2007), microaerophilic (Trojan et al., 2021), able to reduce iron (Coates et al., 1999; Kulichevskaya et al., 2014; Falagán et al., 2017), involved in sulfur cycling (Hausmann et al., 2018; Flieder et al., 2021), able to oxidize atmospheric dihydrogen gas (Giguere et al., 2021), or produce secondary metabolites (Crits-Christoph et al., 2018). Kristensen et al. describe the high diversity and potential involvement of aquatic represent of the phylum *Acidobacteriota* in nitrogen and phosphorous removal along with iron reduction from activated sludge of Danish waste water treatment plants using a MAG-based approach. In doing so, they propose two novel candidate species within the genus *Geothrix*.

While most *Acidobacteriota* have been described as chemoorganoheterotrophic mesophiles, the genus *Choracidobacterium* is one of the few thermophilic and the first and until now sole genus in the phylum whose members perform chlorophyll-dependent phototrophy (Bryant et al., 2007; Tank and Bryant, 2015). Here, Saini et al. report on the isolation of 8 additional *Chloracidobacterium* strains using a defined growth medium. Based on genomic and phenotypic characterization of this extended collection of isolates two further species, “C. aggregatum” and “C. validum”, of the genus within the novel family “Chloracidobacteriaceae”, order *Blastocatellales*, class *Blastocatellia* are proposed. Another thermophilic group of *Acidobacteriota* in this article collection was reported by Ruhl et al. who explore the not yet cultivated “GAL08” group (Ackerman, 2006) from hot spring sediments of Dewar Creek, British Columbia, Canada (Sharp et al., 2014). Based on their findings the GAL08 clade represents a candidate order (*Ca*. Frugalibacteriales) affiliated with the class *Blastotcatellia*; genomic metabolic reconstruction predicts an aerobic heterotrophic metabolism, incomplete denitrification and fermentation. Environmental screening and laboratory enrichment suggest a thermophilic microaerophilic lifestyle for members of this clade.

While growth promoting effects of plant-associated acidobacteria already have been reported earlier (Kielak et al., 2016; Yoneda et al., 2021), Wang et al. for the first time studied animal-associated characteristics of *Acidobacteriota* isolated from a chiton. Using comparative genomics and high-throughput sequencing analyses, they compared one previously described (Fukunaga et al., 2008) and one novel *Acanthopleuribacteraceae* strain, proposed as “Sufidibacter corallicola”. That way, they identified genomic features that indicated these *Acidobacteriota* evolved/adapted to the animal host.

In conclusion, this collection of research articles expanded our knowledge and understanding into (potential) physiological traits of phylum *Acidobacteriota* members from individual acidobacterial strains to whole clades, novel phylogenetic and taxonomic insights, further clues on their ecology, diversity and abundance in selected habitats, and gives further evidence for the environmental relevance of this wide spread though still elusive phylum. Knowledge is expected to continue growing, as demonstrated by the articles in this collection, most likely through the combination of classical microbiological and modern molecular methods.

## Author Contributions

BF organized this Research Topic and drafted the editorial article. All authors listed have made significant, intellectual contributions to organizing, and further developing the topic. All of them contributed to writing the editorial article and approved it for publication.

## Conflict of Interest

The authors declare that the research was conducted in the absence of any commercial or financial relationships that could be construed as a potential conflict of interest.

## Publisher's Note

All claims expressed in this article are solely those of the authors and do not necessarily represent those of their affiliated organizations, or those of the publisher, the editors and the reviewers. Any product that may be evaluated in this article, or claim that may be made by its manufacturer, is not guaranteed or endorsed by the publisher.
